# Sir Alfred Keogh and the R.A.M.C.

**Published:** 1917-08-18

**Authors:** 


### The Hospital

The Workers' Newspaper of Administrative Medicine and Institutiona[l] Life, Administration, National Insurance and Health.

No. 1628, Vol. LXII. SATURDAY, AUGUST 18, 1917. PRICE ONE PENNY

### SIR ALFRED KEOGH AND THE R.A.M.C.

Dispatch, in the business of war, is a great virtue. Yet there are occasions—and present experiences illustrate the truth—when wisdom bids us hasten slowly. No such exhortation, however, applies to the succour of the wounded. Here, at all events, dispatch takes an unchallenged position. Military necessities, or alleged military necessities, have in many wars been responsible for horrible denials in this direction. But in the present struggle the Medical Service has come into its own. The soldiers have been taught that the more efficient this service the more full and capable will be the fighting ranks. The health of the armies will be maintained in these conditions, and a larger proportion of sick and wounded men will be promptly restored to fighting value. The motive so displayed has its hard utilitarian aspect, but war is largely a stern, practical business, and calculations must be framed on this basis. Promptness in care of the wounded has also its individual and sentimental appeal. It strengthens the soldier to see his injured comrades rapidly succoured, and to know that in similar case he himself may count on like help. Of the feelings of the relatives of the fighting men on this subject it is not necessary to speak. They are known in all our homes. Doctors, like their fellow citizens, come within this group, but, in addition, they have to regard the position with a professional eye. They, more than others, know how vital is the difference in the degree of opportunity between promptness and delay. What may with relative certainty be accomplished, given the arrival of the wounded in their hands within a few hours of the battle, becomes impossible when delay complicates the situation. The contrast, put bluntly, is between life and death. The enterprise of modern surgery has much accentuated this position. Its capacity to save life has enormously increased, but it is effective only on conditions. And chief among these conditions is dispatch.

When these truths are remembered there will be high appreciation of the work of the R.A.M.C. and of the forethought and organisation on which this work depends. Promptness in the movement of the wounded to conditions which permit the most skilful and exact surgery has been a governing principle throughout. Every medical officer is imbued with it, and it is urged and emphasised in the highest quarters. Its realisation in practice, too, has gradually become an increasing quantity, and the whole organisation has been adjusted to this end. Of much of the effect nothing is heard outside the individual injured soldier and those to whom he is dear. But some recent facts are known, and they deserve to be put on record. It is a simple statement of actual happenings to say that after several recent battles men wounded on the morning of the fight were safe in London hospitals on the evening of the same day. What this has meant to the men themselves and to their relatives can hardly be put into words. Our medical readers will know what opportunity such an experience has offered to the life-saving and limb-saving capacities of the surgical art. What needs to be emphasised is that triumphs of this order are the outcome of a deliberate policy. They mean sound judgment, forethought, calculation, and the able adjustment of means to ends. Not by some happy accident or fortuitous chance do they arrive. Before they occur in practice they have been planned with a purpose and detailed arrangements have been made to secure them. It is seemly that to those who have accomplished these things should come public gratitude and recognition. Some of our modern critics would appear to think that their whole function is to exploit errors and to exaggerate mistakes. Such a view, we are convinced, is in the most worthy circles regarded with something like contempt. For ourselves, we have never hesitated at plain speech, but the speech has to be attuned to the occasion. Amidst the duties of censure for incompetence and the correction of errors it would be unworthy to fail to find an opportunity to praise famous men and to offer gratitude, and good will to high endeavour spent in the nation's cause.

The particular achievement to which we here draw attention stands on its own merits. But we record it also because it is typical of the spirit of the work which the R.A.M.C. is performing, and because it is an expression of the mind and purpose and ability of those who are directing this service. Rumours, now happily contradicted, have been spread that the Medical Department of the War Office was likely to lose the advantage of its supreme head, and that Sir Alfred Keogh was on the point of retiring. We take the liberty of saying that such an event would be a profound misfortune to the national interest and an occasion of deep regret to all those who care for the wounded soldier and for the health of the Army. The contradiction of the report is a welcome announcement. Difficult times are ahead of the Army Medical Service. The supply of civilian practitioners has practically come to an end. Some arrangement must be made to secure effective co-operation between military and civilian interests. This co-operation will demand knowledge, tact, decision, and sympathy. It is largely because we are satisfied that Sir Alfred Keogh can bring these qualities to the existing situation that we regard this with hopefulness and confidence. He has rendered great service to the nation. But the immediate future presents new responsibilities, and these may be faced with content if Sir Alfred Keogh will accept the burden of direction and control.

